# Using mixed methods to evaluate efficacy and user expectations of a virtual reality–based training system for upper-limb recovery in patients after stroke: a study protocol for a randomised controlled trial

**DOI:** 10.1186/1745-6215-15-350

**Published:** 2014-09-06

**Authors:** Corina Schuster-Amft, Kynan Eng, Isabelle Lehmann, Ludwig Schmid, Nagisa Kobashi, Irène Thaler, Martin L Verra, Andrea Henneke, Sandra Signer, Michael McCaskey, Daniel Kiper

**Affiliations:** Research Department, Reha Rheinfelden, Salinenstrasse 98, 4310 Rheinfelden, Switzerland; Institute for Rehabilitation and Performance Technology, University of Applied Sciences, Pestalozzistrasse, 4301 Burgdorf Switzerland; YouRehab Ltd, Ruetistrasse, 8952 Schlieren, Switzerland; Insitute of Neuroinformatics, University of Zurich and ETH Zurich, Winterthurerstrasse, 8057 Zurich Switzerland; Department of Physiotherapy, Inselspital, Bern University Hospital, Freiburgstrasse, 3010 Bern, Switzerland; Department of Physiotherapy, Queen Margret University, Queen Margaret Drive, Musselburgh, EH21 6UU United Kingdom; Department of Physiotherapy, Clinic Lengg, Bleulerstrasse, 8000 Zurich, Switzerland; Department of Health, Bern University of Applied Sciences, Schwarzstorstrasse, 3007 Bern, Switzerland; Department of Physiotherapy, Buergerspital Solothurn, Schöngrünstrasse, 4500 Solothurn, Switzerland

**Keywords:** Occupational therapy, Physiotherapy, Stroke, Upper-limb function, Virtual reality

## Abstract

**Background:**

In recent years, virtual reality has been introduced to neurorehabilitation, in particular with the intention of improving upper-limb training options and facilitating motor function recovery.

**Methods/Design:**

The proposed study incorporates a quantitative part and a qualitative part, termed a *mixed-methods approach*: (1) a quantitative investigation of the efficacy of virtual reality training compared to conventional therapy in upper-limb motor function are investigated, (2a) a qualitative investigation of patients’ experiences and expectations of virtual reality training and (2b) a qualitative investigation of therapists’ experiences using the virtual reality training system in the therapy setting. At three participating clinics, 60 patients at least 6 months after stroke onset will be randomly allocated to an experimental virtual reality group (EG) or to a control group that will receive conventional physiotherapy or occupational therapy (16 sessions, 45 minutes each, over the course of 4 weeks). Using custom data gloves, patients’ finger and arm movements will be displayed in real time on a monitor, and they will move and manipulate objects in various virtual environments. A blinded assessor will test patients’ motor and cognitive performance twice before, once during, and twice after the 4-week intervention. The primary outcome measure is the Box and Block Test. Secondary outcome measures are the Chedoke-McMaster Stroke Assessments (hand, arm and shoulder pain subscales), the Chedoke-McMaster Arm and Hand Activity Inventory, the Line Bisection Test, the Stroke Impact Scale, the MiniMentalState Examination and the Extended Barthel Index. Semistructured face-to-face interviews will be conducted with patients in the EG after intervention finalization with a focus on the patients’ expectations and experiences regarding the virtual reality training. Therapists’ perspectives on virtual reality training will be reviewed in three focus groups comprising four to six occupational therapists and physiotherapists.

**Discussion:**

The interviews will help to gain a deeper understanding of the phenomena under investigation to provide sound recommendations for the implementation of the virtual reality training system for routine use in neurorehabilitation complementing the quantitative clinical assessments.

**Trial registration:**

Cliniclatrials.gov Identifier: NCT01774669 (15 January 2013)

**Electronic supplementary material:**

The online version of this article (doi:10.1186/1745-6215-15-350) contains supplementary material, which is available to authorized users.

## Background

Virtual reality (VR)–based training is a fast-developing field of rehabilitation and has its origins in the gaming industry. In recent years, VR has been introduced into neurorehabilitation with the intention of improving upper-limb training options and facilitating motor function recovery. *Virtual reality* is defined as the ‘use of interactive simulations created with computer hardware and software to present users with opportunities to engage in environments that appear and feel similar to real-world objects and events’ [1].

So far, few studies have evaluated the efficacy of VR training in chronic stroke rehabilitation [2]. In 2011, Laver *et al*. analysed 19 randomised controlled trials in their systematic literature review, which included only seven RCTs that focused on upper-limb motor function recovery [2]. A meta-analysis of five trials revealed a moderate effect (Cohen’s *d* = 0.53) of VR compared to conventional therapy. However, six different commercial or customised VR training systems were used in the analysed trials. No conclusion could be reached regarding the effect of VR training on grip strength. Furthermore, no recommendations could be suggested regarding dosage, type or programme of the VR training.

Over the past few decades, VR technology and its applications have changed. Besides its potential to trigger external stimulation, it is hypothesized that VR induces use-dependent plastic changes in response to internal stimulation of higher motor cortical areas that recruit the motor memory system, which consists of stored motor programs. This so-called VR-based interactive cognitive intervention is based on the idea that stimulation of the action-processing system in turn activates downstream cortical areas involved in movement execution. There a population of neurons—‘mirror neurons’—is purported to play a key role, as these neurons discharge during both action execution and action observation or imagery [3]. With the recruitment of a widespread movement network normally involved in movement execution, VR-based cognitive therapy offers a potential to specifically promote and/or enhance functional movement recovery. You *et al*. demonstrated important neuroplasticity changes in the primary sensorimotor cortex after VR training in stroke patients [4]. The observed changes were associated with improved locomotion.

Other researchers have shown mirror-like visuomotor activity in the posterior parietal lobe in humans during object-related hand actions [5]. Because task-oriented rehabilitation is known to be beneficial [6], this finding suggests that VR-based cognitive therapy may induce cortical plasticity and promote recovery by using goal-directed arm- and hand movements.

It is hypothesized that a system combining two elements—movement observation with intent to imitate and visualization of mirrored movements of the nonparetic limb—may optimally induce cortical plasticity and functional recovery in both acute and chronic stroke patients. The first element is based on the observation that mirror neurons discharge during goal-directed hand actions and also during observation of another individual performing a similar action. It has been proposed that mirror neurons constitute a vocabulary of hand actions [7]. Their activation leads to recruitment of functionally interconnected cortical structures coupling action execution and observation. The execution–observation system has also been found in humans [3]. Moreover, there is evidence that action observation may facilitate motor activity [3] and induce cortical plasticity [4]. In addition to action execution and observation, mirror neurons and motor-planning areas are known to be activated during voluntary motor imagery, which selectively modulates muscle excitability [4].

In the evaluation of novel technologies developed for neurorehabilitation, it is important to evaluate efficacy and the user’s perspective to enhance successful implementation of the technology. In their literature review, Laver *et al*. suggested assessing patients’ motivation, engagement in the therapy and level of enjoyment [2]. Therefore, a mixed-methods approach will be used in the study proposed here. Together with the randomised efficacy trial it is intended to conduct semistructured patient interviews and focus group interviews with the treating therapists. The interviews will help to provide more information and a deeper understanding of the phenomena under investigation to be used in developing sound recommendations for the implementation of the VR training system for routine use in neurorehabilitation.

The current study includes two parts: (1) a quantitative investigation of the efficacy of virtual reality training compared to conventional therapy in upper-limb motor function are investigated, (2a) a qualitative investigation of patients’ experiences and expectations of virtual reality training and (2b) a qualitative investigation of therapists’ experiences using the virtual reality training system in the therapy setting.

## Methods and design

**Figure 1:**
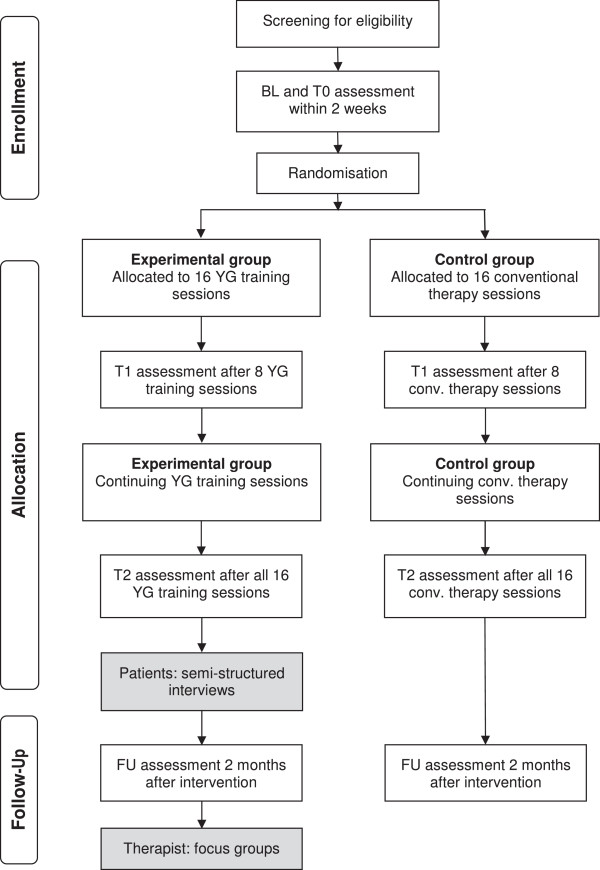
**Study overview.** BL, Baseline; conv., Conventional therapy; T0, Preintervention; T1, Measurement after eight treatment sessions; T2, Measurement event after intervention; FU, Measurement event after 2-month follow-up period; YG, YouGrabber.

This phase III study consists of two parts and envisages a mixed-methods design comprising quantitative and qualitative research methods. The research questions, objectives, hypotheses and methodology of each part are described separately. Figure 1 illustrates the study design.

All study parts will conform to the guidelines of good clinical practice and the Declaration of Helsinki. Ethical approval was obtained from two responsible Swiss ethics committees (ECs): EC Aarau (2012/065) and EC Bern (220/12).

After study implementation, agreed-upon eligibility criteria or therapy content may limit the progress of the study. Therefore, each participating centre or therapist can suggest a modification. However, these modifications can be employed only if all centres agree to accept and apply the modifications and if the responsible ethics committees approve the modifications.

Data collection will be performed in the outpatient centre of a community hospital, an academic hospital and a rehabilitation clinic in the German-speaking part of Switzerland.

### Part 1: randomised controlled trial

#### Study design and measurement events

The first part is designed as a randomised, controlled, assessor-blinded, multicentre trial with two parallel groups and repeated measurement events (MEs). Patients will be evaluated by a blinded assessor on five occasions: twice within 2 weeks at baseline, before intervention start (BL, T0); once after eight treatment sessions (T1); once immediately after the intervention (T2); and once after a 2-month follow-up period (FU).

*Aim*: The aim in the first part is to implement a multicentre, single-blind, randomised controlled trial to investigate the efficacy of VR training compared to conventional therapy. The first part will be implemented at three research sites in the German-speaking part of Switzerland and has two study arms: an experimental group (EG, VR training) and a control group (CG, conventional therapy).

*Research question*: The first part is designed to test the hypothesis that patients after stroke in EG show higher postintervention performance on the Box and Block Test (BBT) compared to CG patients.

*Hypothesis*: It is hypothesised (H_0_) that there will be no group differences after 16 training sessions or after the FU period. It is also hypothesised (H_1_) that there will be a group difference after the 16 training sessions and after the FU period.

#### Patient selection criteria and recruitment

Patients after stroke will be eligible for study participation if they fulfil the selection criteria listed in Table 1.

**Table 1:** **Patient selection criteria**

Inclusion criteria	Exclusion criteria
• ≥6 months after first-ever stroke (ischaemic, haemorrhagic)	• Previous or current other functional deficits of arm and hand motor function not due to stroke
• Able to sit in a normal chair without armrests and without support of the back rest	• Severe cognitive deficits: Mini-Mental State Examination score ≤20
• Persistent motor deficit of arm and hand confirmed by Chedoke-McMaster Stroke Assessment arm subscale level ≥3 and hand subscale level ≥2 (The difference between both subscales has to be two levels or more.)	• Severe visuospatial disorders (for example, severe visual neglect confirmed by LineBisectionTest)
• Able to score at least 1 on the Box and Block Test (main outcome measure)	• History of epileptic seizures triggered by visual stimuli (for example, television, video games) within the past 6 months

The patient recruitment strategy employs different approaches:

1. Patients will be recruited from the clinics’ inpatient or outpatient departments by physicians, therapists and nurses.
2. Patients will be recruited from the clinics’ patient database. Datasets will be screened for study selection criteria by the involved study personnel. If patients are eligible, they will receive a letter describing the study and including patient information. If patients are interested in participating, they can contact the study personnel in the responsible clinic by telephone, postal mail or email.
3. Patients will be recruited via a study information flyer provided on each clinic’s homepage and through patient self-help groups. If patients are interested in participating, they can contact the study personnel in the responsible clinic by telephone, postal mail or email.

#### Randomisation and group allocation

After the patient has received written and oral study information, written informed consent will be obtained from each patient. The procedure will be performed by the clinics’ local study coordinator or the blinded assessors before patient inclusion. Documents (patient information and consent forms) can be obtained from the first author. Patients will be randomly allocated to either the EG or the CG after the second ME (T0). Group allocation will be based on a computer-generated randomisation list (one for all centres), (MATLAB release 2007b; MathWorks, Natick, MA, USA) created by a researcher not involved in the study. The randomisation list will be stored at one clinic’s pharmacy. After the second ME (T0), the treating therapist will contact the pharmacist to disclose the group allocation and the current patient will start with the respective therapy. Group allocation will remain concealed from the independent assessor until study finalisation. Patients and treating therapists will be reminded not to talk with other therapists or participants about group allocations.

As the study is single-blind, the treating therapist will be informed about the patients group allocation. If any unexpected events (severe or nonsevere) occur, the treating therapist will inform the physician on duty and the responsible study organisation personnel to initiate all necessary procedures.

#### Assessor blinding

In intervention studies, it is challenging to keep the assessors blinded. To ensure an objective outcome evaluation, the following procedures will be incorporated:

1. Both interventions will take place in the same room.
2. All therapists can apply both interventions.
3. All patients and therapists will be advised not to talk to the assessor about therapy or training content.
4. Intervention documentation will not be accessible by the assessor.
5. If in any case blinding is uncovered, either a different blinded assessor will perform the assessments or the complete assessment session will be videotaped to allow for an objective evaluation by an assessor from a different participating centre.

#### Study interventions

**Figure 2:**
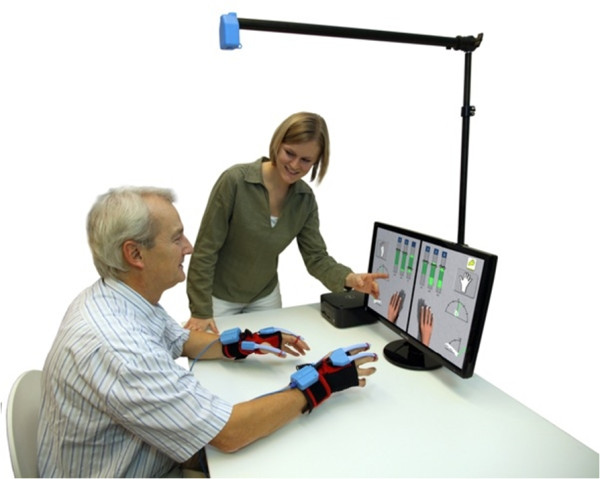
**Virtual reality training system setup (YouGrabber).** The model wears hand gloves with movement sensors attached. The screen displays real-time hand and finger positions.

Both study interventions are described in Table 2 using the Template for Intervention Description and Replication checklist and guide, known as TIDieR [8]. Figure 2 shows the training set up for the experimental group.

**Table 2:** **Description of study interventions based on the TIDieR template** ^**a**^

Item		Experimental group	Control group
1	Brief name	VR training system	Conventional therapy
2	Why	Both interventions will be compared directly in chronic stroke patients for two reasons:	
		1.One-to-one therapy sessions in an adequate amount are limited by health insurance company restrictions.	
		2.If VR technology is used, and YG in particular, patients and therapists will want to know if the treatment effect is the same. If yes, YG could be used to increase the amount of training time with the technology, or it could be recommended as group- or home-based VR training, which would not be the case if YG performed worse.	
3	What: materials	EG patients will sit in front of the VR system (see Figure 2). They will wear hand gloves with attached sensors to measure finger movements of the thumb, index finger, middle finger, wrist (bending, extending) and lower upper limb (pronation, supination). Movements will be displayed on the screen in real time.	No restrictions will be placed on the material used (for example, ADL material, reaching and grasping material). Use of additional electrical or mechanical therapy devices (for example, help arm systems, splints) should be avoided.
4	What: procedures	The VR system has a variety of training applications for different movements and at different levels of difficulty. Therapists can select one of three modes to control the on-screen finger and arm movements: (1) use of the real arm and/or hand movements, (2) mirroring of the real movements of one arm and/or hand and (3) following the movements of one arm and/or hand. The distribution and speed of the appearing objects can be attuned. Furthermore, patients’ movements can be amplified or modulated in the virtual environment to force decreases or increases in training difficulty [9]. After the second VR training session, patients should have tested all training applications and all three modes of finger and/or hand movements. In the remaining 14 sessions, therapists will be asked to select at least 3 training applications for each training session and 2 different movement modes with settings adapted to each patient’s needs.	The therapy content will focus on a task-related upper-limb treatment in a sitting or lying position. Several manual techniques, therapy materials and objects of ADL will be allowed for treatment [10, 11].
5	Who provides	Both study interventions will be provided by experienced physiotherapists or occupational therapists, who will have at least 2 years of professional experience in the field of neurorehabilitation.	
6	How	Both study interventions will be conducted individually in one-to-one sessions.	
7	Where	Both study interventions will take place in the physiotherapy or occupational therapy department of each participating centre.	
8	When and how much	During the 4-week intervention program, patients in both study groups (EG, CG) will receive the same amount of 16 sessions lasting 45 minutes each.	
9	Tailoring	Training and therapy content will be tailored to each patients preferences, the agreed movement aims and the motor function level of each patient.	

^a^ADL, Activities of daily living; CG, Control group; EG, Experimental group; TIDieR, Template for Intervention Description and Replication checklist and guide; VR, Virtual reality; YG, YouGrabber. Items 10, 11 and 12 of the TIDieR template do not apply to this study.

#### Outcome parameters and related outcome measures

Table 3 provides an overview of all outcome measures and measurement events. All data will be collected on a case report form (CRF) that will be stored in a locked cabinet and will not be accessible by the treating therapist. The standardised CRF can be obtained from the first author (CSA).

**Table 3:** **Overview of outcome measures** ^**a**^

Assessment	Abbreviation	Category	Measurement events				
			BL	T0	T1	T2	FU
Box and Block Test*	BBT	Performance measure	X	X	X	X	X
Chedoke-McMaster Stroke Assessment	CMSA	Motor impairment	X	X	X	X	X
Chedoke McMaster Arm and Hand Activity Inventory	CAHAI	Activity (ADL)	X	X	X	X	X
Extended Barthel Index	EBI	Independence	X				
Edinburgh Handedness Inventory	EHI	N/A	X				
Mini Mental State Examination	MMSE	Cognitive assessment	X				
Line Bisection Test	LBT	Neglect assessment	X	X	X	X	X
Stroke Impact Scale	SIS	Impact of stroke on ADL, mobility, emotion, memory, strength, communication	X	X	X	X	X

*Primary outcome measure. ^a^ADL, Activities of daily living; BL, Baseline, FU, Follow-up 2 months after study treatment finalisation; N/A, Not applicable; T0, Preintervention; T1, After eight intervention sessions; T2, Posttest after 16 intervention sessions.

Any patient, who leaves the trial will still be invited to all planned measurement events so that the recover process can be followed. The measurement event will include the same assessments planned for study participants (Table 3).

#### Primary outcome: hand dexterity

Change in hand dexterity between T0 and T2 is the primary outcome of interest. It will be measured with the BBT, which was described by Mathiowetz *et al*. in 1985 [12]. The test is easy to administer and can be quickly performed. It has been used in patients after stroke in patients with multiple sclerosis and traumatic brain injury [13]. Patients will be asked to grasp small wooden cubes and move them from one side of the box to the other as fast as possible within 60 seconds. The BBT is a reliable and valid assessment tool and provides normative data for healthy individuals in age groups ranging from 20 years to older than 75. A change of five or six cubes before and after an intervention seems to be the smallest real difference [14].

#### Secondary outcomes: upper-limb activities of daily living and motor and cognitive function

Upper-limb ADL and motor and cognitive function are secondary outcomes. They will be assessed objectively with the Chedoke-McMaster Stroke Assessment (CMSA), the Chedoke Arm and Hand Activity Inventory (CAHAI) and the Line Bisection Test (LBT) and subjectively with the Stroke Impact Scale (SIS).

The CMSA was developed by Gowland *et al*. in 1995 for the evaluation of physical impairment and activity level of stroke patients [15]. In the present study, we will use the impairment subscales for hand and arm function. Patients will be scored on a seven-point scale (1 = hypoactive or absent muscle reflexes, 7 = no functional impairment detectable anymore, prestroke status) according to seven stages of motor recovery [16]. Additionally, shoulder pain of the affected body side will be evaluated on the same seven-point scale using another subscale. The CMSA has been shown to be a valid and reliable assessment tool [15, 17].

The CAHAI was developed by Barreca *et al*. in 2004 [18–21]. It contains 13 real-life items scored from 1 to 7 (highest score). For example, one item is putting toothpaste on a toothbrush. Scores represent the patient’s relative ability to independently perform stabilisation or manipulation in ADL with the affected upper limb. A score of 1 represents total dependence on another person, and a score of 7 indicates patient independence without time or safety concerns or necessary splints or devices. The test’s reliability and validity have been evaluated [20, 21].

The LBT is a paper-and-pencil test used to evaluate the presence of unilateral spatial neglect (USN) [22]. Patients are asked to mark the centre of a drawn line on paper with a pencil. This is repeated for 18 lines on a sheet of paper. A deviation of more than 6 mm from the centre in the upper and lower 9 lines indicates the presence of USN.

The SIS is a questionnaire comprising questions regarding the impact of stroke on physical function, emotion, memory, communication and social participation. The SIS was developed by Duncan and colleagues in 2001 and has been modified in recent years [23]. The current version, 3.0, consists of eight subscales (strength, hand function, mobility, ADL, emotion, memory, communication and participation) comprising a total of 59 questions that should be administered in a one-to-one interview. Patients can rate the level of their stroke’s impact on a 5-point Likert scale. The reliability, validity and sensitivity to change of this instrument have been evaluated for version 2.0 [24]. Official translations have been produced for 14 languages. The higher the score, the less affected the patient perceives his or her current status to be. A clinically important difference is indicated by changes of 9.2, 5.9, 4.5 and 17.8 points for the strength, ADL, mobility and hand function subscales, respectively [25].

#### Further assessments and evaluations

At study entry, the MiniMental State Examination [26], the Edinburgh Handedness Inventory [27] and the Extended Barthel Index [28] will be used for patient evaluation and descriptive purposes at BL only. Furthermore, patients’ personal information regarding age, gender, marital status, education and profession will be recorded. All currently received therapy sessions will be documented by the examiners during BL assessment. If patients receive another mode of therapy, they will be asked to reduce or suspend it for the duration of the study.

#### Sample size and statistical analyses

On the basis of a study on the efficacy of an earlier study in which the investigators tested the VR system in children with cerebral palsy [29], a power analysis and sample size calculation for the present study were performed with G*Power software version 3.1.5 [30]. In the cited study of children, the BBT (primary outcome measure) showed an effect size (Cohen’s *d*) of 0.98. Assuming a similar effect size for adult stroke patients, we would need (two-tailed test, power = 0.9, significance level *P*-value = 0.05) a total of 46 patients (23 per group). Assuming a dropout rate of 20%, we will recruit a total of 60 patients across the participating centres.

Data will be analysed qualitatively and quantitatively for each patient separately. Changes will be provided as a total number or percentage. Group comparisons between EG and CG will be analysed using analysis of covariance after assessment of normal distribution. We intend to perform an intention-to-treat analyses. If necessary, an additional per protocol analysis will be carried out. Furthermore, subgroup analyses will be performed to investigate any possible study site effects. Patients’ training settings (nominal and interval data) will be analysed with frequencies and percentages to describe therapy content and provide recommendations for further therapy settings.

All study personnel will try to avoid patient dropouts. However, patients can leave the study at any time without reason. Collected patient data until the point of dropout will be included in the final analysis. Missing data will be replaced using two different approaches: (1) with the last available value carried forward method and (2) by adding or subtracting the mean difference of other patients in the respective group. Analysis with both approaches will be performed.

### Part 2

#### Study design and measurement events

It is highly relevant to consider the patients’ and therapists’ perspectives, in particular if new therapy systems will be evaluated regarding their effectiveness. Part 2 comprises two qualitative investigations to explore patients’ and therapists’ experiences with the VR training system. The gained knowledge can be used to improve the training system and to formulate treatment guidelines.

*Aim*: The aim of the investigations is the exploration and description of the patients’ and treating therapists’ experiences and expectations during the intervention with the VR training system. Semistructured interviews will be conducted with patients in EG and focus groups will be performed with therapists, who have trained patients with the VR training system.

*Main research question*: How do patients and therapists experience the intervention with the VR training system?

#### Semistructured interviews with patients

Patients allocated to EG will be offered the opportunity to participate in one semistructured interview after intervention finalisation. They will be informed about and asked for participation during the third or fourth week of the VR training intervention. The face-to-face interview will take between 30 to 60 minutes and will be conducted at the place of the patient’s convenience, either at the treating clinic or at the patient’s home. To provide a high level of privacy, all interviews will take place in a separate and quiet room. Participation will be voluntary, and all interviews will be voice-recorded.

A purposive sampling method will be employed according to the patients’ experienced phenomenon of interest [31]. It is proposed to include a sample of about ten patients to explore and to describe in detail experiences and new knowledge gained in a particular field [32].

#### Interview guide semistructured interviews

Semistructured interviews consist of open-ended questions [33]. The loose structure allows the opportunity to explore and follow an idea deeper without a preimposed interview structure [33]. It is intended to provide an in-depth description of the explored phenomenon [34]. On the basis of existing literature, an interview guide has been developed [35]. The guide divides the interview into three parts (see Additional file 1):

1. Welcoming, information about the interview procedure, questions regarding the rehabilitation process and querying the patient’s current condition.
2. Questions regarding the main research question, such as, What do you think about the VR intervention? How did you feel during the intervention? What were your concerns related to the training system? How did you experience the role of the therapist during the VR training?
3. Interview summary by the interviewer, final remarks of the patient and farewell.

#### Focus groups with therapists

Therapists treating patients with the VR training system will be offered the opportunity to participate in a focus group session that will last from 1 to 2 hours. Therapists will be informed about and asked for participation after they have completed at least one series of patient training with the VR training system, including 16 training sessions or a comparable number of training sessions with different patients. The focus group sessions will be conducted at one of the participating clinics, and three to six therapists will participate in one focus group. Participation will be voluntary, and all interviews will be voice-recorded.

#### Interview guide focus groups

Similar to the semistructured patient interviews, the focus group questions will be open-ended [33]. The guide for the focus groups divides the interview into three parts (see Additional file 2):

1. Welcoming, information about the interview procedure, introduction of focus group participants and taking general questions on the VR training experience.
2. Questions regarding the main research question, such as, How did you experience the patient during the VR training? How did you experience the patient’s motivation? How did you feel during the training with VR?
3. Interview summary of the interviewer, final remarks of the participants and farewell.

Semistructured interview and focus group questions were tested with two patients and therapists beforehand to check for comprehensibility and clarity and were reviewed by the responsible ethics committees.

#### Data analysis of semistructured interviews and focus groups

In addition to the voice-recording of the interviews and focus groups, interviewers will write field notes to describe the interview situation: the way the patient acts during the interview, the patient’s mood and feelings, the course of the interview, unexpected events or statements, and the feelings and impressions of the interviewer. Field notes and the interview and focus group content will provide the basis for the data analysis, which will be based on a descriptive phenomenological approach without data or opinion interpretation [36]. Data analysis will include three steps:

1. TRANSCRIPTION (step 1): Interview content will be transcribed verbatim.
2. CONDENSATION (step 2): Patients’ and therapists’ quotes will be summarised to highlight the main statement.
3. CODING and CATEGORISATION (step 3): On the basis of the thematic analysis, categories and codes will be created to sort patients’ and therapists’ statements [37]. This can be done with the help of a qualitative data analysis tool such as NVivo [38] or ATLAS.ti [39].

#### Overall ethical considerations and reporting of adverse events

The VR training system is a commercial product sold under the name YouGrabber. It uses VR to train upper-limb motor function. It has been available (initially as a beta version) for testing purposes in clinics since 2010 and has been used in rehabilitation institutions and pilot clinical studies with no safety-related incidents. Nevertheless, patients might experience negative emotions or have the impression that they cannot perform the training tasks because of their reduced motor function. It is expected that study participation will take a lot of patients’ time in both groups. The following measures will be utilized in exchange for the patients’ time exposure in the current study:

- Patients will be assessed on a regular basis to evaluate their rehabilitation process.
- Patients will have an intensive therapy schedule with four therapy sessions per week over the course of 4 weeks.
- Patients will have the opportunity to learn about an innovative VR training system.
- Patients can withdraw from the study at any time without giving a reason.

The study will be carried out in accordance with the protocol, with the guidelines for good clinical practice and current national and international valid legal provisions. Any adverse event will be reported to the responsible ethics committee directly and will be mentioned in intermediate and final study reports. The study is registered in the Clinicaltrials.gov trials database with the identifier NCT01774669.

#### Quality control and quality assurance

To achieve and maintain a high quality standard during study preparation and implementation, the following measures will be employed:

- Comprehensive training for all study therapists to use the VR training system before study start.
- Comprehensive training for all study therapists to perform all necessary outcome measures before study start.
- Three refresher training days for VR system training and assessment performance throughout the study
- Study management updates for all centres on a regular basis (monthly).
- Daily telephone and email access for technical support.

Furthermore, up to three monitoring events will be performed for each participating study centre. An authorised person from the sponsor, which is not involved in patient treatment or assessments, will visit the clinics and inspect data handling and patient organisation. Up to five randomly selected patient data files will be inspected.

All study-related patient data will be entered directly onto the anonymised case report forms or assessment scoring sheets. Study-related original patient documents will remain in the responsible clinic and will be archived for 10 years. After that period, patient documents will be destroyed in accordance with the clinic’s data destruction guidelines. Only anonymised electronic data or paper copies will be sent to the study coordinator for data analyses.

Training data collected via the VR training system will be saved in anonymised form under the patient’s study ID, with no other identifying information. Data backup and synchronization will be carried out using a secure, encrypted data transfer and storage framework.

#### Dissemination policy

The study personnel will adhere to an open access policy:

- The trialists intend to publish the study protocol and the study results in international open access journals to provide easy access to the study documents for all interested readers.
- The study is registered in an international open access clinical trial database (Cliniclatrials.gov Identifier: NCT01774669).
- As the study progresses, its methods and preliminary results will be presented at national and international congresses and workshops.
- After study finalisation and data analyses, all study patients will receive a plain language summary of the study results, and the results will be presented in each participating clinic.

Involvement of professional writers is not intended. No restrictions will be placed on the publication of positive or negative results. The study results will be reported in accordance with the guidelines set forth in the 2010 Consolidated Standards of Reporting Trials (CONSORT) [40].

#### Criteria for halting the trial

At present, the commercial YouGrabber system has been used for more than 2 years with over 100 patients in different acute hospitals and rehabilitation clinics. So far, no adverse events have been reported. However, this study will be halted if any of the following criteria are fulfilled:

- More than three EG patients report a sudden onset of or increase in shoulder pain during or just after therapy that is highly likely to be attributable to the use of YouGrabber, and which does not immediately cease after stopping therapy (evaluated as ≤3 on the Chedoke-McMaster Stroke Assessment subscale pain).
- More than 50% of EG patients (minimum of five patients) report severe cybersickness during YouGrabber training which persists after training is halted.
- EG patients show an unexpected decrease in motor function (change of three or more levels in the Chedoke-McMaster Stroke Assessment) indicating a highly significant difference between therapies with YouGrabber compared to conventional therapy.
- Epileptic seizures in at least two patients are induced directly while using YouGrabber.

Patients reporting the criteria mentioned above will be evaluated by the physician on duty and will be assessed and followed up for the originally planned study duration.

#### Unexpected events

Unexpected events will be categorised as severely unexpected events (harmful) and unexpected events (nonharmful). Each event will be documented on a predefined form that is available from the first author (CSA) and will be sent to the overall study coordinator (DK). He will decide if the study insurance provider has to be notified about the event. All events will be reported to the responsible ethics committee independently from the study insurance provider.

## Discussion

The aim of this multicentre trial is to determine the efficacy of a VR-based upper-limb training programme compared to conventional occupational therapy and physiotherapy. Additionally, the users’ perspectives will be evaluated by conducting semistructured patient interviews and therapist focus groups.

In the rapidly evolving VR training industry, it is important to develop a detailed perspective on different aspects of the training interventions used, based on the patients’ and therapists’ perceptions. This goal will be achieved by using a mixed-methods design combining quantitative and qualitative research methods. In particular, from the qualitative data, new hypotheses for further research and adaptations will emerge, including training recommendations and system modifications.

In addition, the knowledge gained in the present study will contribute to the trends described in the review by Laver *et al*. [2]. On the basis of their systematic review of the literature reaching back to March 2010 or earlier, they claimed that the most beneficial patient and VR system characteristics are still undetermined.

The design of a multicentre study offers several advantages and challenges. The shared patient recruitment and treatment methodology provides the opportunity to conduct a sufficiently powered trial to reduce the risk of type I error [41]. Also, therapists and patients from various clinics and different parts of Switzerland can evaluate the VR training options according to personal preferences and local conditions.

In the present study, we decided to use only one randomisation list that will be stored in the pharmacy at one of the three clinics. Therefore, the risk of unblinding the group allocation is reduced, and patient study entry, measurement events and treatment sessions will be under control of one clinic that is responsible for study management.

The planned study is single-blinded only. In general, a sham or placebo intervention in therapeutic disciplines is not always possible. In this study, the treating therapists and patients cannot be blinded to the study group interventions. However, the assessor and the researchers performing the data analyses will be blinded to increase the study’s internal validity and to ensure a low detection bias [42].

Furthermore, each clinic will assign a responsible therapist, who will coordinate patient recruitment and study organisation and conduct therapist training. Regularly scheduled team meetings and study newsletters will help to ensure employment of homogeneous procedures regarding study management, patient treatment and communication with the overall study leader and among all involved clinics.

## Trial status

This study is currently recruiting participants. It is anticipated that the trial will take 18 to 36 months for completion, including all follow-up assessments. The first patient was randomised in February 2013.

## Consent

The authors are thankful to the model in our figure, who illustrates the equipment of the VR training system. Written informed consent was obtained from the model for publication of this manuscript and the accompanying image. A copy of the written consent is available for review by the Editor-in-Chief of this journal.

## Electronic supplementary material

Additional file 1: **Interview guide patient interviews.** (PDF 12 KB)

Additional file 2: **Interview guide therapist focus groups.** (PDF 10 KB)

## Abbreviations

ADL: Activities of daily living
BBT: Box and Block Test
BL: Baseline measurement 2 weeks before intervention start
CAHAI: Chedoke McMaster Arm and Hand Activity Inventory
CG: Control group
CMSA: Chedoke-McMaster Stroke Assessment
EBI: Extended Barthel Index
EG: Experimental group
EHI: Edinburgh Handedness Inventory
FU: Follow-up measurement event
LBT: Line Bisection Test
ME: Measurement event
MMSE: Mini-Mental-State Examination
SIS: Stroke Impact Scale
T0: Measurement event before intervention start
T1: Measurement event after 8 intervention sessions
T2: Measurement event after 16 intervention sessions
VR: Virtual reality.

## References

[1] Weiss P, Kizony R, Feintuch U, Katz N, Selzer M, Cohen LG, Gage F, Clarke S, Duncan P (2006). Virtual reality in neurorehabilitation. Textbook of Neural Repair and Rehabilitation.

[2] Laver KE, George S, Thomas S, Deutsch JE, Crotty M (2011). Virtual reality for stroke rehabilitation. Cochrane Database Syst Rev.

[3] **A holistic approach to stroke care how a therapeutic group was set up to help patients come to terms with the psychological effects of stroke** *Nurs Times* 2002, **98:**33–35.

[4] You SH, Jang SH, Kim YH, Hallett M, Ahn SH, Kwon YH, Kim JH, Lee MY (2005). Virtual reality–induced cortical reorganization and associated locomotor recovery in chronic stroke: an experimenter-blind randomized study. Stroke.

[5] Centers for Disease Control and Prevention (2007). Prevalence of stroke—United States, 2005. MMWR Morb Mortal Wkly Rep.

[6] The Innovative Medicines Initiative (IMI) Strategic Research Agenda 2006.http://ec.europa.eu/research/fp6/pdf/innovative_medicines_sra_final_draft_en.pdf

[7] Aben I, Denollet J, Lousberg R, Verhey F, Wojciechowski F, Honig A (2002). Personality and vulnerability to depression in stroke patients: a 1-year prospective follow-up study. Stroke.

[8] Hoffmann TC, Glasziou PP, Boutron I, Milne R, Perera R, Moher D, Altman DG, Barbour V, Macdonald H, Johnston M, Lamb SE, Dixon-Woods M, McCulloch P, Wyatt JC, Chan AW, Michie S (2014). Better reporting of interventions: template for intervention description and replication (TIDieR) checklist and guide. BMJ.

[9] Eng K, Siekierka E, Pyk P, Chevrier E, Hauser Y, Cameirao M, Holper L, Hägni K, Zimmerli L, Duff A, Schuster C, Bassetti C, Verschure P, Kiper D (2007). Interactive visuo-motor therapy system for stroke rehabilitation. Med Biol Eng Comput.

[10] Luke C, Dodd K, Brock K (2004). Outcomes of the Bobath concept on upper limb recovery following stroke. Clin Rehabil.

[11] Barreca S, Wolf SL, Fasoli S, Bohannon R (2003). Treatment interventions for the paretic upper limb of stroke survivors: a critical review. Neurorehabil Neural Repair.

[12] Mathiowetz V, Volland G, Kashman N, Weber K (1985). Adult norms for the box and block test of manual dexterity. Am J Occup Ther.

[13] Connell LA, Tyson SF (2012). Clinical reality of measuring upper-limb ability in neurologic conditions: a systematic review. Arch Phys Med Rehabil.

[14] Chen HM, Chen CC, Hsueh IP, Huang SL, Hsieh CL (2009). Test-retest reproducibility and smallest real difference of 5 hand function tests in patients with stroke. Neurorehabil Neural Repair.

[15] Gowland C, Van Hullenaar S, Torresin W, Moreland J, Vanspall B, Barreca S, Ward M, Huijbregts M, Stratford P, Barclay-Goddard R (1995). Chedoke-McMaster Stroke Assessment: Development, Validation, and Administration Manual.

[16] Gowland CA (1990). Staging motor impairment after stroke. Stroke.

[17] Gowland C, Stratford P, Ward M, Moreland J, Torresin W, Van Hullenaar S, Sanford J, Barreca S, Vanspall B, Plews N (1993). Measuring physical impairment and disability with the Chedoke-McMaster Stroke Assessment. Stroke.

[18] Barreca S, Gowland CK, Stratford P, Huijbregts M, Griffiths J, Torresin W, Dunkley M, Miller P, Masters L (2004). Development of the Chedoke Arm and Hand Activity Inventory: theoretical constructs, item generation, and selection. Top Stroke Rehabil.

[19] Barreca S, Stratford P, Masters L, Lambert CL, Griffiths J, McBay C (2006). Validation of three shortened versions of the Chedoke Arm and Hand Activity Inventory. Physiother Can.

[20] Barreca SR, Stratford PW, Lambert CL, Masters LM, Streiner DL (2005). Test-retest reliability, validity, and sensitivity of the Chedoke Arm and Hand Activity Inventory: a new measure of upper-limb function for survivors of stroke. Arch Phys Med Rehabil.

[21] Schuster C, Hahn S, Ettlin T (2010). Objectively-assessed outcome measures: a translation and cross-cultural adaptation procedure applied to the Chedoke McMaster Arm and Hand Activity Inventory (CAHAI). BMC Med Res Methodol.

[22] Plummer P, Morris ME, Dunai J (2003). Assessment of unilateral neglect. Phys Ther.

[23] Duncan PW, Wallace D, Studenski S, Lai SM, Johnson D (2001). Conceptualization of a new stroke-specific outcome measure: the Stroke Impact Scale. Top Stroke Rehabil.

[24] Duncan PW, Wallace D, Lai SM, Johnson D, Embretson S, Laster LJ (1999). The Stroke Impact Scale Version 2.0: evaluation of reliability, validity, and sensitivity to change. Stroke.

[25] Lin KC, Fu T, Wu CY, Wang YH, Liu JS, Hsieh CJ, Lin SF (2010). Minimal detectable change and clinically important difference of the Stroke Impact Scale in stroke patients. Neurorehabil Neural Repair.

[26] Folstein MF, Folstein SE, McHugh PR (1975). “Mini-Mental State”: a practical method for grading the cognitive state of patients for the clinician. J Psychiatr Res.

[27] Oldfield RC (1971). The assessment and analysis of handedness: the Edinburgh Inventory. Neuropsychologia.

[28] Jansa J, Pogacnik T, Gompertz P (2004). An evaluation of the Extended Barthel Index with acute ischemic stroke patients. Neurorehabil Neural Repair.

[29] van Hedel HJA, Wick K, Eng K, Meyer-Heim A: **Improving dexterity in children with cerebral palsy: preliminary results of a randomised trial evaluating a glove based VR-system.** *Proceedings of the 2011 International Conference on Virtual Rehabilitation (ICVR): 2011 June 27–29; Zurich*127–132. doi:10.1109/ICVR.2011.5971872

[30] Faul F, Erdfelder E, Lang AG, Buchner A (2007). G*Power 3: a flexible statistical power analysis program for the social, behavioural, and biomedical sciences. Behav Res Methods.

[31] Starks H, Trinidad SB (2007). Choose your method: a comparison of phenomenology, discourse analysis, and grounded theory. Qual Health Res.

[32] Kvale S, Brinkmann S (2009). InterViews: Learning the Craft of Qualitative Research Interviewing.

[33] Britten N (1995). Qualitative interviews in medical research. BMJ.

[34] Finlay L (2011). Phenomenology for Therapists: Researching the Lived World.

[35] Hughes AM, Burridge J, Freeman CT, Donnovan-Hall M, Chappell PH, Lewin PL, Rogers E, Dibb B (2011). Stroke participants’ perceptions of robotic and electrical stimulation therapy: a new approach. Disabil Rehabil Assist Technol.

[36] Speziale HS, Carpenter DR (2007). Qualitative Research in Nursing: Advancing the Humanistic Imperative.

[37] Braun V, Clarke V (2006). Using thematic analysis in psychology. Qual Res Psychol.

[38] Wiltshier F: **Researching with NVivo.** *Forum Qual Soc Res* 2011.,**12**(1)**:**

[39] Mühlmeyer-Mentzel A: **The logical structure of data in ATLAS.ti and its advantage for grounded theory studies.** *Forum Qual Soc Res* 2011.,**12**(1)**:**

[40] Moher D, Hopewell S, Schulz KF, Montori V, Gøtzsche PC, Devereaux PJ, Elbourne D, Egger M, Altman DG (2010). CONSORT 2010 explanation and elaboration: updated guidelines for reporting parallel group randomised trials. BMJ.

[41] Munro BH (2005). Statistical Methods for Health Care Research.

[42] Higgins JP, Altman DG, Gotzsche PC, Juni P, Moher D, Oxman AD, Savovic J, Schulz KF, Weeks L, Sterne JA (2011). The Cochrane Collaboration’s tool for assessing risk of bias in randomised trials. BMJ.

