# Supplementary material for: Using mixed methods to evaluate efficacy and user expectations of a virtual reality–based training system for upper-limb recovery in patients after stroke: a study protocol for a randomised controlled trial
Source: Trials. 2014 Sep 6;15:350. doi: 10.1186/1745-6215-15-350 (PMC4167274; doi:10.1186/1745-6215-15-350)
Supplement: Supplementary file 1 — Additional file 1: Interview guide patient interviews. (PDF 12 KB) [file 13063_2014_2223_MOESM1_ESM.pdf]

Additional file 1: Interview guide patient interviews.

Sequence	Topic	Question
**Introduction**	About the stroke	Welcome statement<br>When was your stroke?<br>How do you feel today?<br>What is the main problem for you today with your arm?
	Prior experience with virtual reality	What prior experiences have you had with virtual training?<br>Do you have any prior knowledge of virtual training?<br>How comfortable did you feel with computer systems prior to your stroke?
**Main part**	Virtual reality training system	What have you experienced with the YouGrabber?
	Start of the intervention	How was your first experience with YouGrabber?<br>What did you expect from the first session?<br>How did you feel during the first session?<br>What did you expect from the first intervention?
	Virtual reality training system	What were your apprehensions concerning that intervention?<br>What did you like/dislike in the intervention?<br>What was easy/difficult at the beginning with the device?<br>What was the main experience with YouGrabber?<br>How did you like it?<br>How, in your opinion, can YouGrabber help you to achieve your goals?<br>What do you think about the YouGrabber intervention?<br>How did you feel during and after the intervention?<br>What did you expect at the end of the training program?<br>Have your expectations changed?<br>What were your concerns at the end of the training session?<br>Did your concerns change?
	Motor function	Did something (arm function) change over the training period? (in the therapy, in daily life, at home?)
	Virtual reality training system	What was easy/difficult at the end of the training program?
	Therapist role	What was the therapist role?
	Virtual reality training system	What would you have changed, if anything?
**Closing questions**	Virtual reality training system	Any comments or do you want to add something?<br>If you could change anything in the intervention or the device, what would it be?
	Interview	Final summary from the researcher and the participant.<br>How did you feel about the interview? (stressful, agreeable, difficult)
