# Supplementary material for: Using mixed methods to evaluate efficacy and user expectations of a virtual reality–based training system for upper-limb recovery in patients after stroke: a study protocol for a randomised controlled trial
Source: Trials. 2014 Sep 6;15:350. doi: 10.1186/1745-6215-15-350 (PMC4167274; doi:10.1186/1745-6215-15-350)
Supplement: Supplementary file 2 — Additional file 2: Interview guide therapist focus groups. (PDF 10 KB) [file 13063_2014_2223_MOESM2_ESM.pdf]

Additional file 2: Interview guide therapist focus groups.

Sequence	Topic	Question
**Introduction**	Virtual reality training system	What are your general experiences on the YG System?
**Main part**		What was your expectation before first YG Training?
		How did you experience the patients on the YouGrabber?
		What developments did you notice at the patients?
		What changes did you remark in the handling of the YouGabber?
		How did you as a therapist feel during the YG Trainings?
	Virtual Reality	What risks do you see in the treatment with VR?
		What chances do you see in the treatment with VR?
		How do you appraise future posibilities of VR in stroke rehabilitation?
**Closing questions**		Do you have something to complete?
		How do you think about the interview?
